# Viscoelasticity assessment of tumoral skin with the use of a novel contact-free palpation methodology based upon surface waves

**DOI:** 10.1038/s41598-022-23483-4

**Published:** 2022-11-04

**Authors:** A. Bergheau, J.-L. Perrot, R. Vargiolu, H. Zahouani

**Affiliations:** 1 Laboratory of Tribology and System Dynamics (LTDS), UMR 5513 CNRS, ECL-ENISE, Ecole Centrale de Lyon, University of Lyon, 36 Avenue Guy de Collongue, 69134 Ecully, France; 2 Centre Hospitalier Universitaire de Saint-Etienne, Saint-Priest-en-Jarez, France

**Keywords:** Biomedical engineering, Basal cell carcinoma, Melanoma

## Abstract

The ensuing pilot investigation sheds new light on characterizing tumoral and non-tumoral human skin mechanical properties that will not only assist the dermatologist’s diagnosis but also could constitute the creation of an Artificial Intelligence database for upcoming research. A modern, non-invasive, and contact-free methodology—UNDERSKIN—was developed, and hinges upon Fourier transform computations that permit the analysis of surface wave dispersion with a specific skin inversion model and viscoelastic model. It yields a detailed look at how particle movements of the medium propagate throughout its near sub-surface, hence a novel knowledge of the mechanical responses of skin tumors. The research results display the tumors’ viscoelastic responses alongside their respective healthy skin outcomes for each skin layer as well as the dermatologist’s touch analysis. Although dermatologists are capable of sensing and having a fair overall assessment of what they are palpating, they are unable heretofore to quantify it and inform where the firmness or softness derives from, which it is necessary to be acquainted with so as to perform an accurate diagnosis, prognosis, treatment, future surgery, and teledermatology.

## Introduction

In the medical examination field, palpation is one of the classic exploration techniques of the human body and is part of the in-vivo diagnostic method^1^. Besides having a nodding acquaintance with its ancient history, palpation techniques have been employed since antiquity. Palpation is the process of utilizing one's hands to inspect the body, especially while perceiving/diagnosing a disease or illness. Frequently performed by a healthcare practitioner, it is the process of sensing an object in or on the body to determine its size, shape, firmness, and/or location. Palpation is essential for physical examination—touch is as essential as sight. Physicians developed great aptitude in palpating problems underneath the body surface, permitting them to detect tiny aspects that untrained people would not. Yet, mastery of anatomy and much practice are required to achieve a high skill level.

In addition, this vision considerably benefited from in-vivo modern technological advances such as dermatoscopy, confocal microscopy, bi-photonic microscopy, LC-OCT^2–4^ (DAMAE MEDICAL, Paris, France), and Raman spectroscopy. However, even though touch is systematically utilized while skin examination is seeking surface irregularities—keratosis actinic detection—or profound palpation so as to ascertain whether the tumor is soft or not, quantifiable measurements remain nonexistent. For example, Scleroderma, commonly characterized by sclerosed infiltration of the skin, is quantified by dint of Rodnan score, which is qualitative. In addition, lipoma—a subcutaneous tumor—is soft during palpation, whereas Malherbe calcified epithelioma is hard. Both retain different amounts of ulsus infiltration; therefore, dermatologists seek the tumor’s mechanical responses to assess their diagnosis, hence cancer prognosis.

Physical asperities are challenging to palpate, making additional, quantifiable measurement medical tests more than necessary to gain a comprehensive understanding of these. Indeed, many pathologies involve tissue structural changes resulting in modifying their mechanical properties such as elasticity. This palpation ought to be supplemented by modern techniques that would convey to the physician an indication of the elasticity embedded in the multi-layered structure of biological tissues—e.g., skin—in which a paucity of equipment remains. Elastography, for example, is a relatively novel technology and entered the clinic arsenal mostly in the last decade. The most prominent techniques apply ultrasound or magnetic resonance imaging (MRI) to create the stiffness map and an anatomical image for comparison. However, the use of these technologies is designed for profound tissue. They are therefore unlikely to measure mechanical responses of the skin's outermost layers^5–8^. Therefore, a gap must be filled, and a specific device designed to analyze the mechanical properties of the thinnest layers of skin must be developed. Indeed, outcomes from a modern digital palpation assessment equipment could not only inform about where the firmness/softness of skin tumors originate from, but it would also aid in their early detection, thus helping to precise/quantify the tumor’s invasive character. Prior to solving the endeavor that constitutes the mechanical response of skin cancers, the multi-layered structure of skin ought to be primarily analyzed and comprehended.

## Results

### Sensitive methodology: ex-vivo testing

This experiment consists in laying isolated porcine Stratum Corneum on a soft substratum: alginate. An adequate amount must be set to ensure no reflections/refractions induced by the glassware it is deposited on during data acquisition. Not only does the softness of this mixture considerably aid in removing these interferences, but it also creates a behavioral variation compared to the response of the Stratum Corneum. 1-cm depth of Alginate proved to be suitable in that case. Hereupon, two measurements are carried out with UNDERSKIN to assess the sole Alginate response and the Stratum Corneum atop upon it.

**Figure 1:**
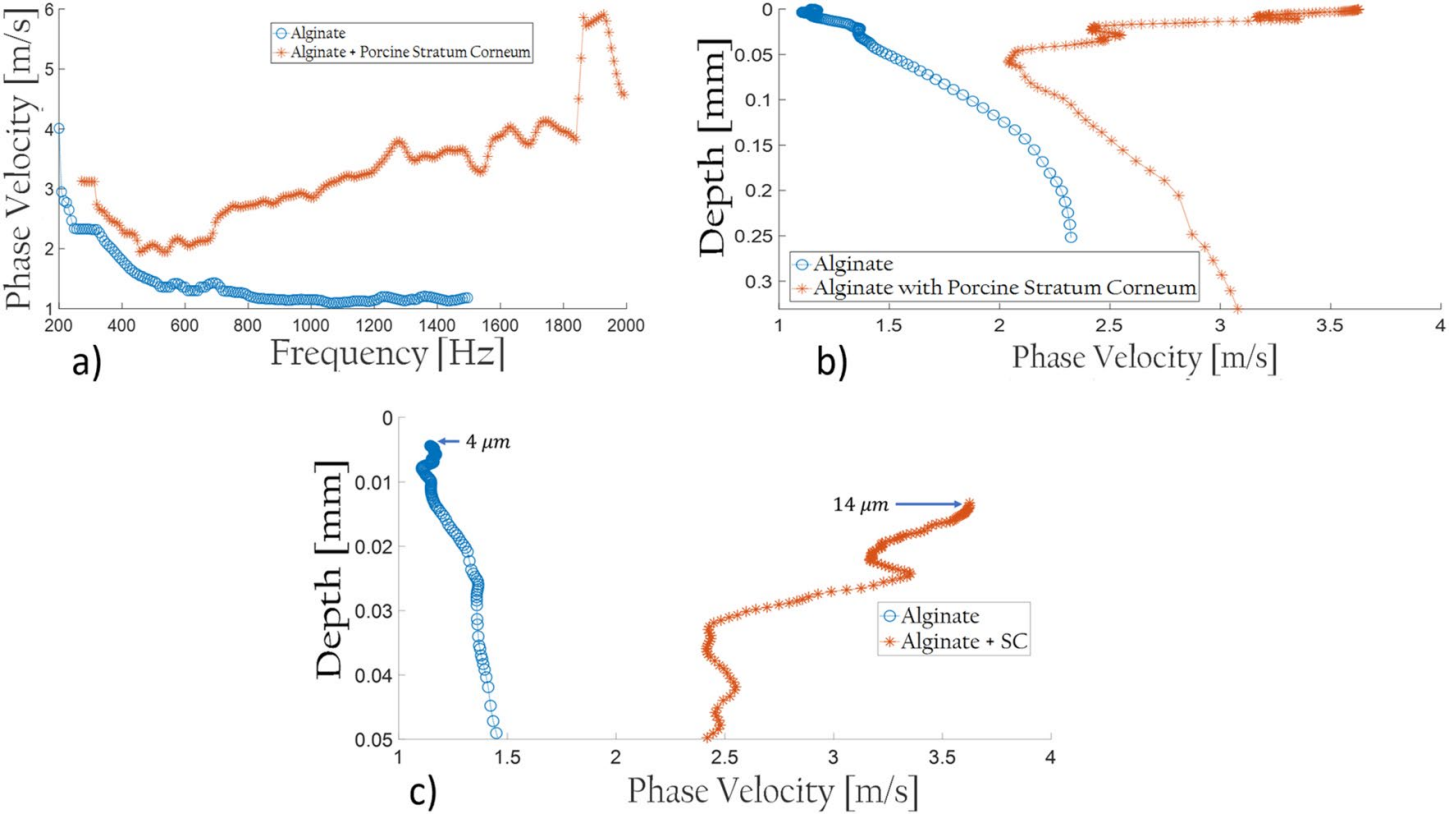
(**a**) Dispersion curve outcomes of Alginate and Alginate with Porcine Stratum Corneum atop. (**b**) Velocity profiles resulted from the dispersion curves with the inversion model applied. (**c**) Close-up on the velocity profiles resulted from the dispersion curves with the inversion model applied.

First and foremost, the two dispersion outcomes in Fig. 1a illustrate two distinguished behaviors after 600 Hz, although the response of these two is not dissimilar from 200 to 500 Hz. One remark is to be fastened upon these results, though: the dispersion outcome of the sole Alginate resembles a seismic one. The thin overlayed water that remained at the surface made the material softer atop, hence the phase velocities propagating slower in higher frequencies than in the lower ones. Besides, some sort of dual-slope can be ascertained in the two-layered structure, whereas a single one is observed for the sole Alginate. It demonstrates the device to be of extreme sensitivity to detect the presence of the thin layer on the grounds of the phase velocity response changing around 600 Hz. Moreover, the inversion model, assessed in the Online Method section, is applied accordingly to the dispersion results, as observed in Fig. 1b. Although the behavior of both velocity profiles below 50 microns is quite identical—being cognizant of a velocity offset induced by the Stratum Corneum—a substantial increase is discerned in the first 50 microns regarding the two-layered material. Furthermore, zooming in on the first measured microns (Fig. 1c), both curves do not originate from the same point in depth. Not only is this caused by the inversion model employed, but a difference of 10 microns is discovered by dint of it—in which it corresponds to the thickness of the Stratum Corneum applied. This value is acceptable considering that LC-OCT^2^ (Line-field Confocal Optical Coherence Tomography) evaluated it to be 15 microns.

### Pathology palpation assessment

The whole following pilot investigation has been carried out at the dermatology unit at Saint-Etienne’s academic health center. UNDERSKIN technology is henceforth part of and utilized in this department amid advanced skin imaging technologies such as LC-OCT^2^. All methods were conducted in accordance with relevant guidelines and regulation, approved by the ethics committee “Terre d’Ethique”, and all the subjects signed an informed consent.

On a side note, all measurements undertaken with UNDERSKIN have been performed in one single direction with 5 measurements per tumor.

#### Papillomatous nevus

A male patient, 60 years of age, had a papillomatous nevus located on the forearm. After UNDERSKIN signal processing was achieved, the velocity outcome (Fig. 3a) illustrated high values in the outermost region. By all means, it reflects the hardness of the pathology’s crust, as it is confirmed in Fig. 3b. Moreover, a hump is witnessed around 2 mm deep, certainly conveying information about the tumoral structure. Indeed, as confirmed by the dermatologist during palpation Fig. 3c exposes the papillomatous nevus viscosity parameter to be greater than the surrounding healthy skin.

#### Angioma

**Figure 2:**
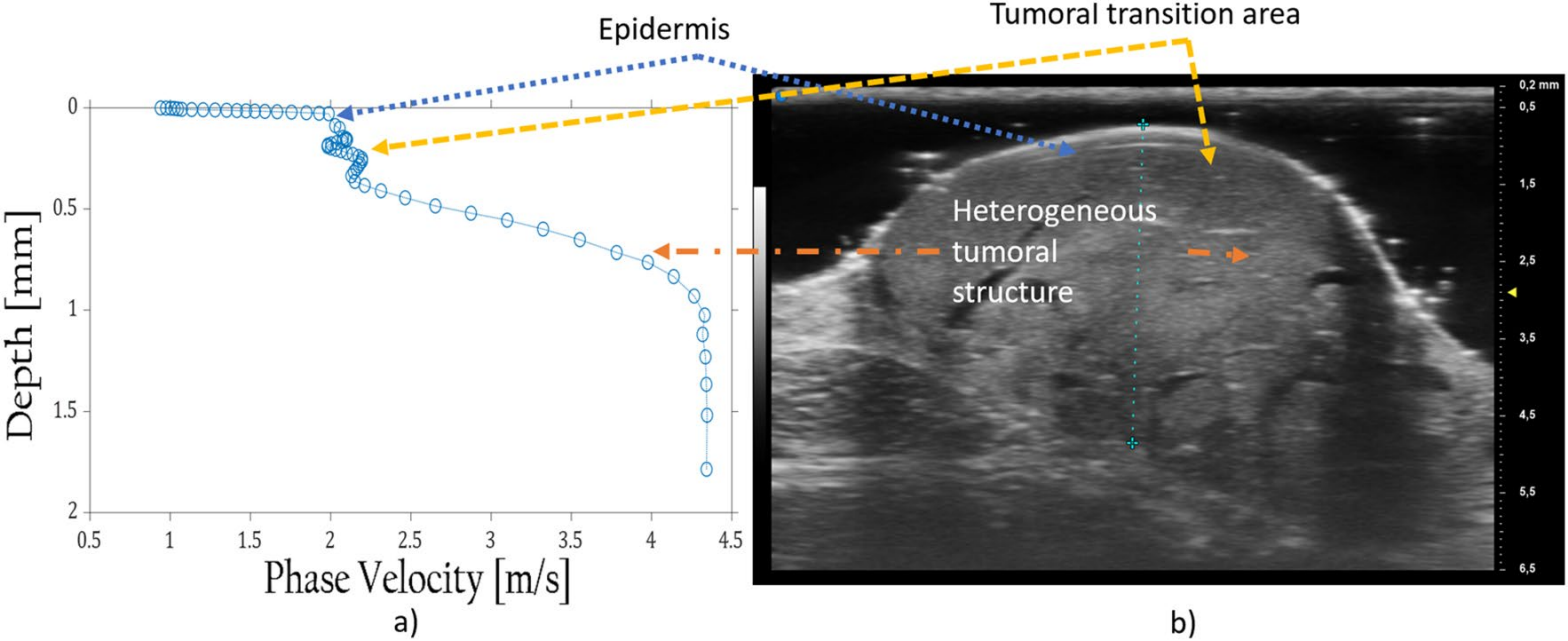
Cross correlation between Angioma velocity profile (**a**) and mode-B image (**b**).

**Figure 3:**
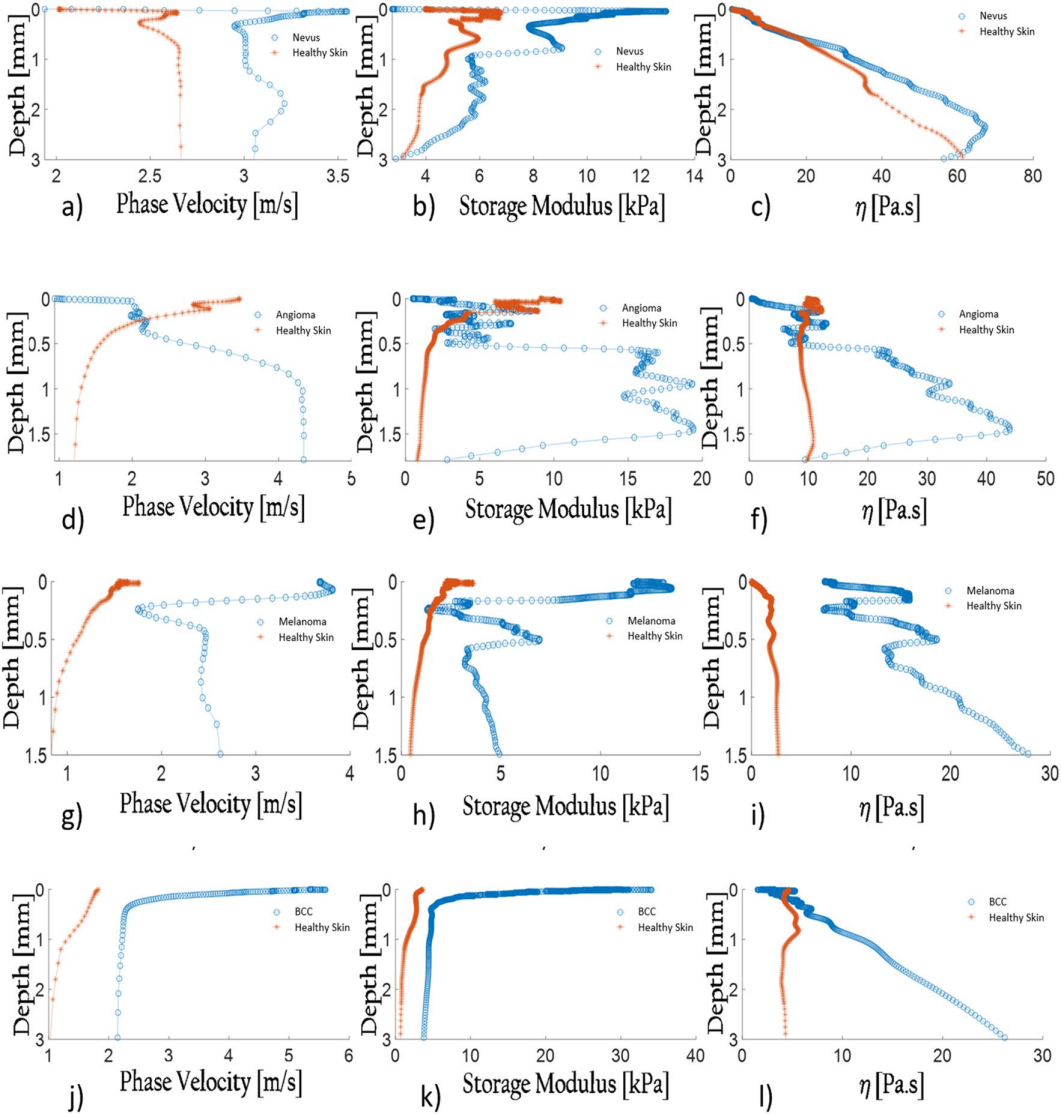
(**a**) Angioma velocity profile in comparison with adjoing skin velocity profile. (**b**) Angioma storage modulus profile in comparison with adjoing skin storage modulus profile. (**c**) Angioma viscosity parameter profile in comparison with adjoing skin viscosity parameter profile. (**d**) Melanoma velocity profile in comparison with adjoing skin velocity profile. (**e**) Melanoma storage modulus profile in comparison with adjoing skin storage modulus profile. (**f**) Melanoma viscosity parameter profile in comparison with adjoing skin viscosity parameter profile. (**g**) BCC velocity profile in comparison with adjoing skin velocity profile. (**h**) BCC storage modulus profile in comparison with adjoing skin storage modulus profile. (**i**) BCC viscosity parameter profile in comparison with adjoing skin viscosity parameter profile. (**j**) Papillometous nevus velocity profile in comparison with adjoing skin velocity profile. (**k**) Papillometous nevus storage modulus profile in comparison with adjoing skin storage modulus profile. (**l**) Papillometous nevus viscosity parameter profile in comparison with adjoing skin viscosity parameter profile.

Once the blast of air has hit the angioma and signal processing is achieved, the velocity outcome (Fig. 2a) differs from the common one discovered in healthy skin (Fig. 3d). Applying our inversion model to this dispersion result leads to satisfactory accuracy (Fig. 2b) in comparison with the mode-B image captured of it. It illustrates a soft material atop with a roughness inside. A transition area can be observed between 100 and 500 microns as well, which both measurement techniques are able to detect. Below 500 microns, the medium resembles a heterogeneous tissue.

The viscoelastic model yields great results compared to healthy skin. Indeed, measurements were carried out on the tumor, and next to it. Figure 3d illustrates how the phase velocity behaviors differ from one another. It can relate to the structural differences between tumoral skin and non-tumoral skin in which the mechanical response is doubtlessly dissimilar. Not only does the storage modulus (Fig. 3e) of the top layer expose ample variations—ranging from 1 to 5 kPa for the tumoral environment against 5–10 for the healthy skin, but the viscosity parameter $\eta$ (Fig. 3f) also sheds biomarkers’ new light as it is of extreme viscosity—ranging from 10 to 45 Pa s for the angioma and around 10 Pa s for the healthy skin.

#### Melanoma

In the following analysis, a 54-year-old woman had a tumor that was soft, friable, and hemorrhagic spontaneously during palpation. Histological examination proved it to be an achromic melanoma—3.6 mm’s thick—ulcerated with a high proliferation index. The examination employing polarized dermatoscopy—FotoFinder Medicam 1000, Germany, Bad Birnbach—with $\times 30$ optical magnification revealed a pattern associated with a bicolor (red and whitish veil) homogeneous structure and ulcerations.

Following the UNDERSKIN measurement and after applying the viscoelastic model to the velocity profile ascertained, relevant outcomes ensue, peculiarly when compared to healthy skin. Figure 3g illustrates how the phase velocity in-depth differs from one another. It can relate to the structural differences between tumoral skin and non-tumoral one in which the mechanical response is undoubtedly distinguishable. Not only does the storage modulus (Fig. 3h) display immense variations in terms of the outermost layer response—ranging from 5 to 15 kPa for the tumoral environment against 2 to 4 for the adjoining cutaneous tissue, but the viscosity parameter $\eta$ (Fig. 3i) also illustrates novel biomarker insights as it is extremely evolving—ranging from 10 to 30 Pa s for the melanoma and 0 to 4 for healthy skin.

#### Basal-cell carcinoma: BCC

A female patient of age 84 had a BCC located on the cheek with a typical surface appearance and a hypoechoic heterogeneous structure measured to be 1 mm thick according to the VisualSonic ultrasound (Fujifilm vevo MD HF, Toronto, Canada)^9^. Histological examination confirmed the diagnosis in which an invasive subtype had been assessed. Moreover, the tumor was hard during palpation, which is characteristic of this cancer type.

After data acquisition and the signal was processed, the velocity result (Fig. 3j) alluded to a healthy skin response yet having an uncommon skewed velocity increase in the top layer region. In all likelihood, it reveals the stiffness of this pathology. Employing the inversion model to this result, it illustrates a rigid material around the outermost region with a hypoechoic heterogeneous inside between 0.2 and 2 mm.

According to the viscoelastic model outcomes on both explored sites—tumoral and non-tumoral zones—Fig. 3k, in fact, reveals the hardness of the tumor in the first 500 microns in depth. Moreover, although the phase velocity and the storage modulus do not vastly vary between 1 and 3 mm, the loss modulus induces significant changes. Indeed, the hypoechoic heterogeneous tumoral structure produces a rise of the viscosity parameter (Fig. 3l), ranging from 10 to 28 Pa s against 5 Pa s for healthy skin.

## Discussion

Literature is scarce regarding tumoral skin’s viscoelastic parameters. The objective herein was not to claim the discovery of the proper viscosity or shear modulus of tumoral skin, but rather to have values both homogeneous and akin to them, and to assess the feasibility of a pilot study that will bode well for further research. The collected data in Table 1 display the tumors’ viscoelastic responses alongside their respective healthy skin outcomes for each skin layer as well as the dermatologist’s touch analysis. As a result, applying the viscoelastic model to pathology measurements conveyed novel insights for the dermatologist. Not only did the outcomes confirm diagnosis while employing human palpation, but the dermatologist is now able to quantify them and inform where the firmness or softness stems from. In addition, these results could nourish AI algorithms to create a database in what, all of which is essential to be enlightened so that it will stand the practitioner in good stead for forthcoming diagnosis, prognosis, treatment, and surgery.

**Table 1:** Viscoelastic outcomes of the measured melanoma, Angioma, BCC, and Papillomatous Nevus including storage modulus relative percentage calcutated over the tumor and healthy skin results $\frac{{G}^{\mathrm{^{\prime}}}\left(tumor\right)- {G}^{\mathrm{^{\prime}}}\left(Healthy\,Skin \right)}{{G}^{\mathrm{^{\prime}}}\left(Healthy\,Skin\right)}$, viscosity relative percentage calculated over the tumor and healthy skin results $\frac{\eta \left(tumor\right)- \eta \left(Healthy\,Skin \right)}{\eta \left(Healthy\,Skin\right)}$, and dermatologist’s palpation assessment.

	${G}^{^{\prime}} (\mathrm{kPa})$	Relative %	$\eta (\mathrm{Pa s})$	Relative %	Dermatologist palpation assessment
**0–200 µm**					
Papillomatous nevus	13	100	9.8	− 2	Soft
Healthy skin	6.5		10		
Angioma	4	− 60	4.1	− 60	Firm, yet less than the melanoma
Healthy skin	10.1		10.3		
Melanoma	15	275	15.2	334	Firm
Healthy skin	4		3.5		
BCC	34	794	6	3.4	Extremely firm
Healthy skin	3.8		5.8		
**200 µm–1 mm**					
Papillomatous nevus	9	50	23	9.5	Soft
Healthy skin	6		21		
Angioma	17	467	25.4	125	Firm, yet less than the melanoma
Healthy skin	3		11.3		
Melanoma	6	200	17.3	394	Firm
Healthy skin	2		3.5		
BCC	5	28.2	10	69.5	Extremely firm
Healthy skin	3.9		5.9		
** > 1 mm**					
Papillomatous nevus	4.4	15.8	65	8.3	Soft
Healthy skin	3.8		60		
Angioma	17	750	34.4	212	Firm, yet less than the melanoma
Healthy skin	2		11		
Melanoma	5	525	22.1	591	Firm
Healthy skin	0.8		3.2		
BCC	4	300	22	323	Extremely firm
Healthy skin	1		5.2		

Moreover, dermatologists’ sight has considerably increased over the years by virtue of skin imaging advances. Consequently, enhancing the practitioner’s touch is nothing less than indispensable, and the preliminary study undertaken proved UNDERSKIN to be earmarked as a valuable assistance device to gain objective knowledge of tumoral and non-tumoral skin mechanical behavior. Besides that, it ought to be noted that OCE^10^ (Optical Coherence Elastography) is a novel methodology that brings forth useful data concerning mechanical characterizations of the skin layers, and it would be of great interest to compare OCE outcomes with our methodology results in future research. Furthermore, ex-vivo assessments such as bio-printed organs and biopsies, or in-vivo analyses such as skin analysis for cutaneous pathological tumors’ viscoelastic anisotropy, the healing of a wound, knowing whether the viscoelastic properties of healthy skin return after tumor removal, and comprehending by dint of numerical simulation, whether or not, dispersion occurs due to the layered structure or its viscoelastic properties shall comprise upcoming research and undoubtedly vouchsafe novel insights for the digital palpation world.

## Online method

### Introduction

Besides offering essential protection from environmental threats^11^, other serendipitous characteristics lie in the uppermost layer of skin—stratum corneum—which can be utilized for other purposes. Over the past decade, not only did the tape stripping technique for removal of the outermost layer arouse great interest apropos of its skin barrier function^12^, but this also can be seen as a powerful tool to observe the mechanical behavior of human skin layers. This section introduces a novel, non-invasive, and contract-free technology dubbed UNDERSKIN which employs surface waves to assess mechanical properties of skin beneath the surface. Undoubtedly, assessing its mechanical behavior is of utmost relevance. There are numerous ways to characterize soft tissue material, such as Ultrasound-Elastography^13^, Acoustic-Radiation-Force Impulse Elastography, and Magnetic Resonance Elastography^6^. However, all these methods utilize a tracking in-vivo shear wave propagation algorithm, making it challenging to achieve decent resolutions for the human top skin layers^14–16^: Stratum Corneum, Epidermis, and the Dermal–Epidermal Junction. In addition, a gel is in contact between the skin and the probe—doubtlessly altering the mechanical response of the cutaneous tissue. A modern, non-invasive, and contact-free methodology is proposed utilizing the computation of surface wave dispersion. Indeed, the principles of surface waves have been employed in geophysics for decades to assess the elastic properties of soils and map them. A myriad of techniques have been developed, such as Spectral Analysis of Surface Waves (SASW) and Multiple Analysis of Surface Waves (MASW). Based upon Fourier transform computations of the recorded wavefield u(x,t), these permit the analysis of the surface wave dispersion by calculating phase velocities as a function of frequency^17^. Therefore, the dispersion curve outcome alongside the attenuation values of the surface wave will shed new light on how the particle movement of the medium propagates throughout its near sub-surface. Incorporating the tape stripping approach into that process yields key variations which can be evaluated and utilized as an inversion model. Consequently, it illustrates a detailed visual of the mechanical behavior across the thinnest layers of skin.

### UNDERSKIN device

**Figure 4:**
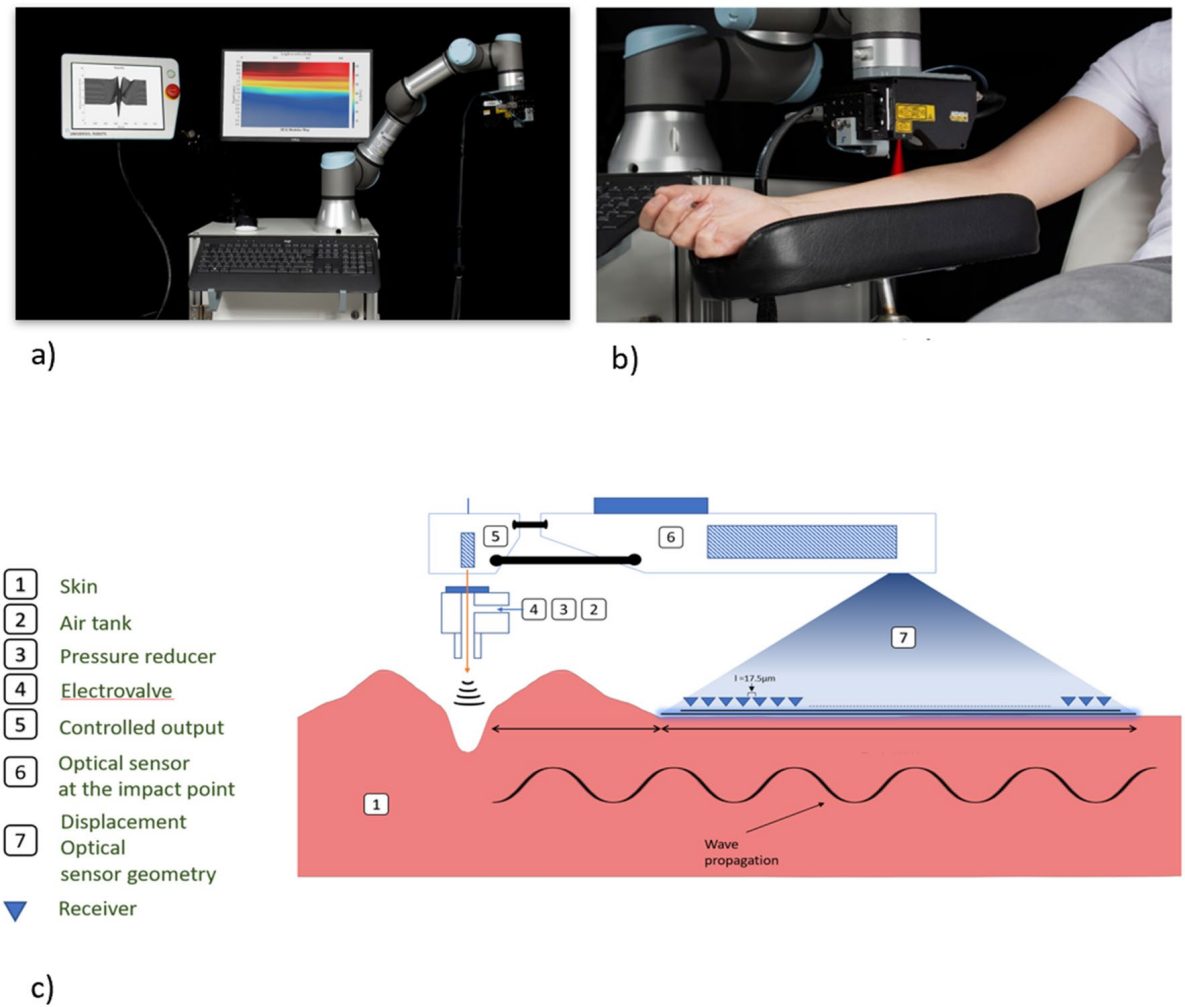
(**a**,**b**) UNDERSKIN device. (**c**) Recorded wavefield measurement principle.

Envision a precise, painless blast of air disrupting the skin’s surface so that it creates ripples. UNDERSKIN records and measures these ripples with a low-power laser optical displacement sensor. The laser line, whose length L = 7 mm, comprises 400 receivers spaced of dx = 17.5 µm and is located at an offset distance ${x}_{1}$ of 0.3 mm from the source—the airflow. The air pressure is set at 3 bars, the sample rate ${f}_{s}$ at 8 kHz, and the impact time at 7 ms. These elements—airflow and sensor—are attached to a multi-jointed robotic arm. It will aid in attaining any human body zone that requires to be measured. It extends and rotates. See Fig. 4 a,b&c for details.

**Figure 5:**
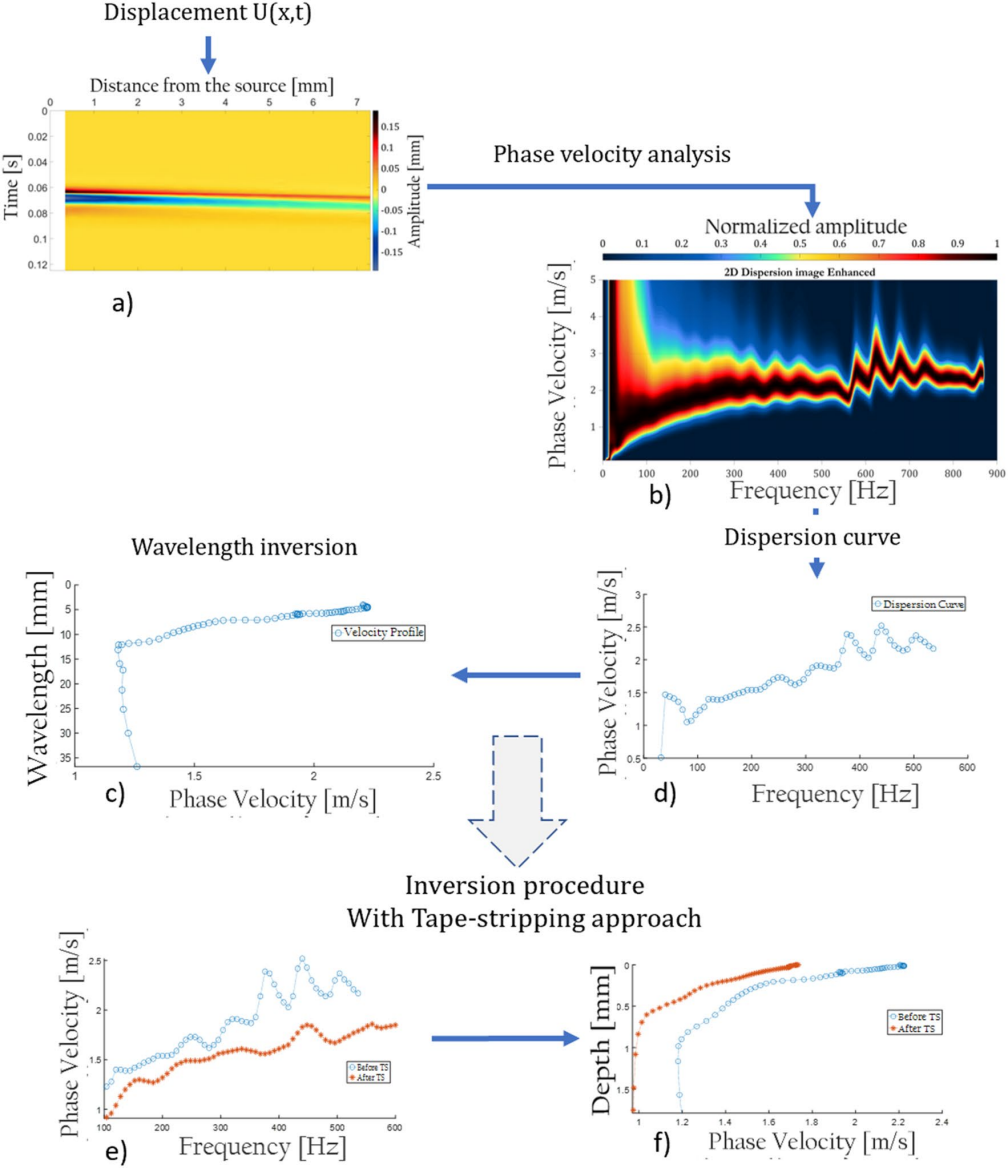
(**a**) Recorded wavefield; displacement as a function of time and the distance from the source—blast of air. (**b**) Dispersion image resulting from MASW algorithm. (**c**) Dispersion curve extracted from the dispersion image. (**d**) Phase velocity as a function of the wavelength. (**e**) dispersion curves outcome before and after tape-stripping so as to elaborate the inversion model. (**f**) Phase velocity as a function of the depth employing the established inversion model.

This records the displacement as a function of the time and the space domain at each receiver location of the sensor line (Fig. 5a). The dispersion analysis of the wavefield outcome will ensue.

### Signal processing

#### Phase-shifting algorithm

The phase-shifting methodology—also known as the wavefield transformation method—was initially introduced by Park in 1998^18^. The phase-shift algorithm is a wave transformation technique to obtain a phase-velocity spectrum—dispersion image (Fig. 5b)—based on a multichannel-impulsive shot gather^18,19^.

Utilizing the phase-shift method, the dispersion properties of all sorts of waves—body and surface waves—contained in the recorded data are visualized in the frequency-phase velocity/transformed energy domain. Different modes of surface waves are identified by their frequency content as well as their characterizing phase velocity at each frequency. Noise sources, that is body waves and reflected/scattered waves, are likewise recognized by their frequency content and move out across the receiver array^20^. The required Rayleigh wave dispersion curves are extracted from the dispersion image for further analysis. Noise is habitually automatically eliminated in this process^21,22^.

This method can be divided into 3 steps:

- Fourier transform and amplitude normalization
- Dispersion imaging
- Extraction of dispersion curves

#### Dispersion curve and velocity profile

The extraction of one or several dispersion curves from the dispersion image outcome rests upon the energy content of the recorded wavefield^23,24^. The fundamental mode dispersion characteristics are generally of primary interest as the most utilized inversion methods operate only with the fundamental mode dispersion curve^20,21,25^. However, a specific inversion model for skin depth assessment was adequately elaborated and will be enlarged upon the next subheading.

A phase velocity is extracted for each frequency according to the maximum amplitude $A({V}_{r},f)$ ascertained, see Fig. 5c. Hereupon, each ${V}_{r}, f$ pair value will deliver the corresponding wavelength $\lambda$ utilizing the dispersion relation (1) as follow:

1 $$\lambda \left( f \right) = \frac{{V_{r} \left( f \right)}}{f}$$

Then, a velocity profile can be plotted with the wavelength as a function of the phase velocity (Fig. 5d).

#### Inversion model and penetration depth

Traditionally, the maximum depth of investigation ${z}_{max}$ varies with the site and the source of the tremor. It is then related to the longest surface wave wavelength that is obtained during data acquisition ${\lambda }_{max}$ adding a factor to it. This $\alpha$ factor is empirically commonly adopted between one-third and one-half^19,26,27^.

2 $${z}_{max}= \alpha .{\lambda }_{max}$$

However, lower frequencies that propagate more rapidly than higher frequencies constitute, by all means, one of the Rayleigh wave’s specificities. It accounts for the behavioral structure of soils or ocean waves. It is quite the contrary of what is resulting from the skin. A lamb-wave pattern is ascertained in which it describes the behavior of a thin overlayed structure going from a stiffer layer to a less stiff one^28^. Additionally, we observe quite a difference in the frequency range between a dispersion image of soils and a dispersion image of the skin. Therefore, a classic Rayleigh wave inversion model—the link between the wavelength and the depth—cannot be applied accordingly to investigate the skin’s near sub-surface. A brand-new model ought to be developed.

In fact, the tape stripping (TS) technique appeared to be a great asset to observe the penetration depth of the surface wave generated by the blast of air. Ordinarily, tape stripping is a less invasive method to collect skin samples^12^. Tape strips are small plastic discs with an adhesive side; they stick to the skin surface and the epidermis's components adhere to the plastic when removed^11^. The consecutive removal permits deeper levels of the stratum corneum to be accessed. This technique induces barely any discomfort yet leaves a momentary red mark. However, it does not require skin preparation and causes neither bleeding nor scarring^11^. The idea herein is to undertake a measurement with UNDERSKIN before and after consecutive tape strips to evaluate how the dispersion of the surface wave varies as a function of what has been removed—$\Delta depth$. Additionally, LC-OCT^2^ will be measuring the depth removal with great accuracy, strengthening the $\alpha$ factor outcome. The velocity difference $\Delta {V}_{r}(f)$ in between the two dispersion results—before and after TS in Fig. 5e—yields a wavelength difference $\Delta \lambda (f)$ as a function of the frequency range. Therefore, the link between $\Delta \lambda (f)$ and $\Delta depth$ conveys new insights on the value of $\alpha$ for frequencies above 100 Hz. In addition, knowing the value of that factor for the low part of the spectrum—from 10 to 100 Hz—from the literature (approximately 0.3), an exponential fit is applied to connect dots, producing then the first inversion model—See Fig. 5f to observe the inversion model applied.

To complete the elaboration of the inversion model, a panel of 5 healthy volunteers was part of the TS experiment. To begin with, UNDERSKIN is measuring the velocity profile for every single volunteer, and this will be set as a reference. Besides, LC-OCT^2^ accompanies this process to determine the thickness of the outermost layer for each volunteer. Then, the principle is to remove the horny layer owing to 20 consecutive tape strips to assess it comparatively to the measurement undertaken beforehand. D-squame tape is utilized in this investigation in which it consists of adhesive disks with a standardized 14 mm diameter. Then, UNDERSKIN assesses a new velocity profile alongside the thickness assessment realized with LC-OCT^2^ afterward. The velocity and wavelength difference engendered by the tape stripping lead to a $\alpha$ factor value for each subject. As a result, this factor value is approximated to 0.02 according to this investigation.

### Viscoelastic model

The phase velocity is the sole elasticity marker of the skin thus far, and it can be converted to the shear velocity allowing the computation of the complex shear modulus. Indeed, this complex shear modulus can facilitate the characterization of soft tissues such as skin. The real part of the complex modulus is known as the storage modulus and has been examined extensively as a biomarker for diagnosis and disease staging^29,30^. Nevertheless, orthodox surface wave approaches frequently utilize shear storage modulus derived hinged upon the propagation velocity of the surface wave—commonly employing model-based approximations while omitting the influence of surface wave attenuation^31–33^. In other words, elastography techniques regard the tissue as a pure elastic one^34^ ($E=3\rho {V}^{2}$). Yet, skin consists of 60% of water and this methodology does not pay heed to the viscosity component. It is, regardless, known from recent SWEI methods in bulk media that the shear attenuation and shear loss modulus can be utilized as additional biomarkers^29^. Characterization of surface wave attenuation and loss modulus may add additional insights into the early diagnosis and staging of skin diseases^29^. In this pilot study, we compute shear wave attenuation and model-independent complex modulus from surface wave velocity and attenuation values^29^. Hereafter, modes of surface waves displacement fields can be written as Eq. (3).

3 $${\widetilde{u}}_{m}\left(x,\omega \right)=\sum_{m}{A}_{m}(x,\omega ){e}^{i(\omega t-{k}_{m}\left(\omega \right)x)}$$

where $\omega$ stands for the angular frequency, ${k}_{m}$ for the wave number and ${A}_{m}$ for the amplitude of ${m}^{th}$ mode of the surface wave. Once the surface wave is induced by the blast of air, the term ${A}_{m}(r,\omega )$ can be expressed as a function of the impact source and the propagation distance $x$ (4)^29^.

4 $${\widetilde{u}}_{m}\left(x,\omega \right)=\sum_{m}{I(\omega )R}_{m}(\omega )\frac{{e}^{-{\beta }_{m}(\omega )}}{\sqrt{x}}{e}^{i(\omega t-{k}_{m}\left(\omega \right)x+{\varphi }_{0}(\omega ))}$$

$I(\omega )$ is the amplitude spectrum, ${R}_{m}(\omega )$ the response of the skin, ${\beta }_{m}(\omega )$ the attenuation for the ${m}^{th}$ mode, $\sqrt{x}$ the diffraction factor, and ${\varphi }_{0}(\omega )$ the phase spectrum of the signal. However, a single mode—the fundamental mode—is considered in that study. Equation (4) can be written as (5)^29^.

5 $$\widetilde{u}\left(x,\omega \right)={I(\omega )R}_{m}(\omega )\frac{{e}^{-{\beta }_{x}(\omega )}}{\sqrt{x}}{e}^{i(\omega t-{k}_{x}\left(\omega \right)x+{\varphi }_{0}(\omega ))}$$

Already knowing the phase velocities of the surface wave, we herein utilize (5) to determine the attenuation ${\beta }_{x}(\omega )$ by separating the amplitude and phase components of Eq. (5). Therefore, ${\beta }_{x}(\omega )$ becomes:

6 $${\beta }_{x}\left(\omega \right)x=-\mathrm{ln}\left(\left|\widetilde{u}\left(x,\omega \right)\right|\right)-\frac{1}{2}\mathrm{ln}\left(x\right)-ln({I\left(\omega \right)R}_{m}\left(\omega \right))$$

Henceforth acquainted with the attenuation computation, the complex wavenumber ${k}_{R}$ can be formulated as:

7 $${k}_{R}=\frac{\omega }{{V}_{R}(\omega )}-i{\beta }_{R}\left(\omega \right)$$

It is used, in that case, to derive the complex shear wavenumber ${k}_{shear}$ of the medium. The Rayleigh wave dispersion equation in a viscoelastic media is given by:

8 $$4{{k}_{R}}^{3}\delta -\left({{k}_{shear}}^{2}-{{2k}_{R}}^{2}\right)-{{k}_{shear}}^{4}=0$$

With $\delta =\sqrt{{{k}_{R}}^{2}-{{k}_{shear}}^{2}}$, utilizing (8), ${k}_{shear}$ can be ascertained in terms of shear wave phase velocity as (9).

9 $${k}_{R}=\frac{\omega }{{V}_{R}(\omega )}-i{\beta }_{R}\left(\omega \right)$$

Consequently, in linear and isotropic viscoelastic material, the storage and loss modulus, respectively ${G}^{^{\prime}}$ and $G^{\prime\prime}$, are related to the complex shear wavenumber ${k}_{shear}$ ascertained by dint of Eq. (9) and the Rayleigh wave dispersion equation^29^. Therefore, where $\omega$ stands for the angular frequency, $\rho$ being the medium density, ${V}_{shear}$ the shear velocity, and ${\beta }_{shear}$ the attenuation of the shear wave, the shear storage and loss modulus can be written as follow:

10 $${G}^{^{\prime}}=\rho {\omega }^{2}\frac{{\left(\frac{\omega }{{V}_{shear}\left(\omega \right)}\right)}^{2}-{{\beta }_{shear}(\omega )}^{2}}{{\left({\left(\frac{\omega }{{V}_{shear}\left(\omega \right)}\right)}^{2}+{{\beta }_{shear}(\omega )}^{2}\right)}^{2}}$$

11 $$G^{\prime\prime} = 2\rho \omega^{2} \frac{{\left( {\frac{\omega }{{V_{shear} \left( \omega \right)}}} \right)\beta_{shear} \left( \omega \right)}}{{\left( {\left( {\frac{\omega }{{V_{shear} \left( \omega \right)}}} \right)^{2} + \beta_{shear} \left( \omega \right)^{2} } \right)^{2} }}$$

These constitutes, by all means, additional biomarker insights regarding skin analysis. Besides, the parameter $\eta$—derived from 11—appends one to this, and it is homogeneous to the medium viscosity owing to the loss modulus.

12 $$\eta \left( \omega \right) = \frac{{G^{\prime\prime}\left( \omega \right)}}{\omega }$$

### Phantoms and validation

Testing the viscoelastic model on phantoms is vital prior to employing it on the intricate soft tissue that is skin. Two single-layer viscoelastic silicones with different viscoelastic properties have been created by the world-renown French dressing company URGO and assessed for this pilot investigation. Silicon 1 and Silicon 2 have respectively a storage modulus of 4.6 kPa and 3.9 kPa with a viscosity equal to75 Pa s and 63 Pa s. However, they utilized classic DMA (Dynamic Mechanical Analysis) testing to reveal viscoelastic parameters. Both techniques—DMA and UNDERSKIN—do not measure identical ones as they are two different methodologies; yet the objective is to ascertain the same estimation. In addition, phantoms’ viscoelastic parameter outcomes have resulted from low-frequency DMA testing (10 Hz), whereas UNDERSKIN had measured the response from 50 to 200 Hz.

UNDERSKIN results present identical estimations on the storage modulus and viscosity alike. Ten measurements with exact configurations were performed to ensure repeatability. The values of viscoelastic parameters for each experiment, their standard deviations (SD), and mean values were calculated. Silicon 1 and Silicon 2 have respectively a storage modulus of 4.99 kPa with 0.19 SD and 4.16 kPa with 0.16 SD, and a viscosity equal to 84.7 Pa s with 15.4 SD and 66.6 Pa s with 7.39 SD (Supplementary information S1).

## Supplementary Information

Supplementary Information.

## Data Availability

All data generated or analyzed during this study are included in this published article and its supplementary information files S1.
